# The Rcs-Regulated Colanic Acid Capsule Maintains Membrane Potential in *Salmonella enterica* serovar Typhimurium

**DOI:** 10.1128/mBio.00808-17

**Published:** 2017-06-06

**Authors:** Jasmine M. Pando, Joyce E. Karlinsey, Jimmie C. Lara, Stephen J. Libby, Ferric C. Fang

**Affiliations:** a Department of Microbiology, School of Medicine, University of Washington, Seattle, Washington, USA; b Department of Laboratory Medicine, School of Medicine, University of Washington, Seattle, Washington, USA; UCLA School of Medicine

**Keywords:** *Salmonella*, biofilms, colanic acid, exopolysaccharide, extracytoplasmic stress, proton motive force

## Abstract

The Rcs phosphorelay and Psp (phage shock protein) systems are envelope stress responses that are highly conserved in gammaproteobacteria. The Rcs regulon was found to be strongly induced during metal deprivation of *Salmonella enterica* serovar Typhimurium lacking the Psp response. Nineteen genes activated by the RcsA-RcsB response regulator make up an operon responsible for the production of colanic acid capsular polysaccharide, which promotes biofilm development. Despite more than half a century of research, the physiological function of colanic acid has remained elusive. Here we show that Rcs-dependent colanic acid production maintains the transmembrane electrical potential and proton motive force in cooperation with the Psp response. Production of negatively charged exopolysaccharide covalently bound to the outer membrane may enhance the surface potential by increasing the local proton concentration. This provides a unifying mechanism to account for diverse Rcs/colanic acid-related phenotypes, including susceptibility to membrane-damaging agents and biofilm formation.

## IMPORTANCE

Colanic acid is a negatively charged polysaccharide capsule produced by *Escherichia coli*, *Salmonella*, and other gammaproteobacteria. Research conducted over the 50 years since the discovery of colanic acid suggests that this exopolysaccharide plays an important role for bacteria living in biofilms. However, a precise physiological role for colanic acid has not been defined. In this study, we provide evidence that colanic acid maintains the transmembrane potential and proton motive force during envelope stress. This work provides a new and fundamental insight into bacterial physiology.

## INTRODUCTION

Transmission of the food-borne pathogen *Salmonella enterica* requires survival of the bacterium in the environment. The cell envelope forms a permeability and structural barrier that maintains cellular homeostasis and is essential for environmental persistence. Five regulatory systems sense and respond to extracytoplasmic stress: the CpxAR and BaeSR two-component systems (TCSs), the σ^E^ alternative sigma factor, the Psp (phage shock protein) response, and the Rcs (regulator of capsule synthesis) phosphorelay system (1–6).

Expression of the Rcs system is observed in response to osmotic shock, growth on a solid surface, or exposure to β-lactam antibiotics (6–9). Effectors of the innate immune system, including cationic antimicrobial peptides (CAMPs), complement, and lysozyme, can also induce the Rcs system (10, 11). The Rcs response is initiated by autophosphorylation of the RcsC sensor kinase and proceeds via phosphotransfer by the RcsD protein to the RcsB response regulator (12–14). The Rcs system also includes RcsF, an outer membrane lipoprotein that acts upstream of RcsC (15). The phosphorylated RcsB response regulator can activate the transcription of downstream genes either as a homodimer or as a heterodimer with the auxiliary regulator RcsA (16), which is unstable because of degradation by the Lon protease (17). RcsBA and RcsB homodimers regulate distinctive subsets of genes. RcsA-RcsB-regulated genes are primarily involved in exopolysaccharide (EPS) production and include the 19-gene colanic acid capsular operon and the *yjbEFGH* operon, which encodes the biosynthesis of a distinct EPS (18, 19). RcsB is required for expression of the Rcs response, and increased RcsB expression can compensate for the absence of RcsA with regard to capsular synthesis (14). Genes regulated by RcsB independently of RcsA include *ftsZ*, *osmC*, and *rprA* (20–22).

The Rcs system was first identified by its role in the transcriptional regulation of colanic acid biosynthesis in *Escherichia coli* (23, 24). Although colanic acid was discovered more than half a century ago (25), its physiological function has remained poorly defined. Colanic acid is composed of glucose, galactose, glucuronic acid, and fucose and forms a highly negatively-charged capsule (25, 26). Colanic acid capsule production is not required for systemic *Salmonella* infection in mice (27–29). In contrast to many EPS capsules, colanic acid does not protect against phagocytosis by polymorphonuclear leukocytes (PMNs) or from killing following PMN uptake (26) and confers only minimal resistance to the bactericidal actions of serum complement (10, 26). Adherence of uropathogenic *E. coli* to T84 colonic epithelial cells is impaired by the presence of colanic acid (26). Collectively, these observations do not suggest a primary role for colanic acid in *Salmonella* pathogenesis. Moreover, the production of colanic acid is increased at lower temperatures, consistent with an environmental function (7).

Biofilms are utilized by bacteria to persist in many environmental niches and during chronic infections (30). The ability to form biofilms has been observed in numerous *Salmonella* isolates from environmental, clinical, food, and animal sources (31). Colanic acid capsule has been shown to contribute to biofilm formation in *E. coli* and *Salmonella* (31, 32). Colanic acid has been reported to confer resistance to environmental stresses, including hyperosmolarity, acid pH, desiccation, oxidative stress, and extreme temperatures (33–35). The *yjbEFGH-*encoded EPS (18) is less well characterized but appears to contribute to resistance to hyperosmotic stress (36) and is not required for biofilm formation (8).

Although the individual extracytoplasmic stress responses comprise largely discrete subsets of genes, inactivation of one response can lead to the compensatory expression of others (37–39). In the present study, we observed dramatic induction of the colanic acid capsular regulon following metal deprivation of an *S. enterica* serovar Typhimurium mutant lacking the Psp response. In *Salmonella*, the Psp response is required for virulence in mice expressing natural-resistance-associated macrophage protein 1 (Nramp1) (40), a proton-dependent phagosomal divalent metal transporter (41, 42). Nramp1 enhances host resistance to intracellular pathogens by limiting metal availability within the phagosomal compartment (42, 43). *Salmonella* competes with Nramp1 by expressing energy-dependent metal transport systems (43). By maintaining membrane bioenergetics, the PspA response allows *S. enterica* serovar Typhimurium to acquire essential metals despite the presence of Nramp1 (40). As the Psp response has been shown to preserve proton motive force (PMF) during extracytoplasmic stress (38, 44, 45), we evaluated a possible role for the Rcs stress response and colanic acid capsule biosynthesis in PMF maintenance.

## RESULTS

### EPS production is enhanced in metal-restricted *pspA* mutant *S.* Typhimurium.

**FIG 1:**
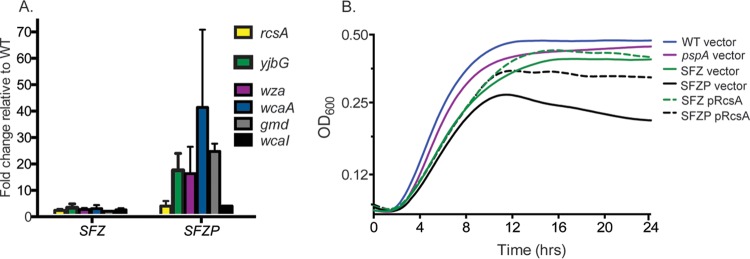
RcsA and EPS genes are induced in SFZP mutant *Salmonella* during metal restriction. (A) Results of qPCR assays performed with cDNA obtained from cultures grown in chelated LB for 2 h. Absolute qPCR values were normalized to the bacterial housekeeping gene *rpoD* and are expressed as the fold change over the WT. Mean qPCR values from three biological replicates ± the standard deviation are shown. (B) Growth curves of strains in LB with 550 μM dipyridyl at 37°C.

The Psp system facilitates metal uptake by *S.* Typhimurium transport systems, including SitABCD, MntH, and ZupT (40). Mutant strains deficient in metal transport exhibit impaired growth following treatment with the chelator 2,2′-dipyridyl (40). In previous studies, the introduction of a *pspA* (P) mutation into an *S.* Typhimurium strain lacking the ABC transporter SitABCD (S), the Fe^2+^ transporter FeoB (F), and the ZIP family permease ZupT (Z) resulted in cell death following treatment with dipyridyl, indicating that the Psp system is necessary for cell survival during metal deprivation (40). To obtain mechanistic insights into the mechanism of cell death, a microarray analysis was performed to analyze the transcriptional response of SFZP (*sit feo zup psp*) mutant *S.* Typhimurium treated with dipyridyl for 2 h. As expected, induction of the Psp operon in an SFZP mutant was observed in the absence of the negative regulator PspA (see Table S1 in the supplemental material). Expression of the *pspA* gene could still be detected in the *pspA* deletion mutant, as the oligonucleotide probe contains sequences outside the deleted region. In addition to the *psp* genes, the most strongly induced loci were those comprising the colanic acid capsule operon (see Table S1), regulated by the RcsA-RcsB heterodimer (16). Other genes in the Rcs regulon, including *rcsA* and the *yjbEFGH* operon (18, 46), were also strongly induced. The induction of *rcsA*, *yjbG*, and genes from the colanic acid capsule operon was confirmed by quantitative PCR (qPCR) analysis (Fig. 1A) and observed only under conditions of iron depletion (see Fig. S1). As an SFZP mutant exhibited induction of the auxiliary regulator *rcsA*, we determined whether an *rcsA*-expressing plasmid could restore growth in chelated medium. Wild-type (WT) or mutant strains containing either a vector control or pRcsA were grown in chelated Luria-Bertani (LB) medium, and growth was monitored by measurement of optical density at 600 nm (OD_600_) (Fig. 1B). As previously observed (40), the viability of an SFZP mutant declined after 12 h of growth. The *rcsA*-expressing plasmid allowed the SFZP mutant to reach a higher cell density and eliminated the decline in viability at 12 h, indicating that the induction of the Rcs response and EPS production following metal deprivation of SFZP mutant *S.* Typhimurium is adaptive.

10.1128/mBio.00808-17.6 TABLE S1  Genes induced ≥4-fold during the growth of a *Salmonella* SFZP (*sit feo zup psp*) mutant in LB with 625 μM dipyridyl. Download TABLE S1, PDF file, 0.4 MB. Copyright © 2017 Pando et al. 2017 Pando et al. This content is distributed under the terms of the Creative Commons Attribution 4.0 International license.

10.1128/mBio.00808-17.2 FIG S1  Growth in LB does not induce expression of *rcsA* or the colanic acid capsule operon. qPCR was performed with cDNA obtained from cultures grown in LB for 2 h. Absolute qPCR values were normalized to the bacterial housekeeping gene *rpoD* and expressed as the fold change over the WT. Mean qPCR values from three biological replicates ± the standard deviation are shown. The *pspD* gene is highly expressed in the SFZP mutant because of the absence of the negative regulator PspA and is shown as a positive control. Download FIG S1, PDF file, 0.4 MB. Copyright © 2017 Pando et al. 2017 Pando et al. This content is distributed under the terms of the Creative Commons Attribution 4.0 International license.

### The Psp response maintains membrane integrity under metal-restricted conditions.

**FIG 2:**
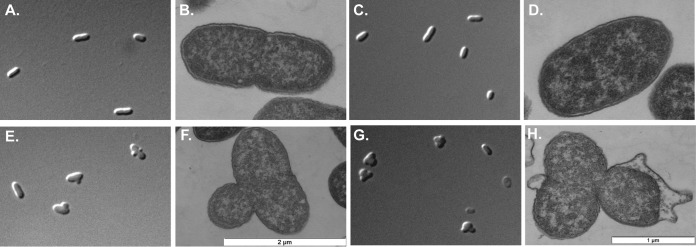
*S.* Typhimurium *sitA feoB zupT* metal transport mutants lacking the phage shock response lose cell membrane integrity. *Salmonella sitA feoB zupT* (SFZ) (A to D) or *sitA feoB zupT pspA* (SFZP) (E to H) mutants were diluted 1,000-fold in LB with 625 μM dipyridyl and incubated for 3 h (A, B, E, F) or 4 h (C, D, G, H). Representative DIC (A, C, E, G) and TEM (B, F, D, H) images are shown. DIC, ×100, oil immersion. B and F, TEM at ×20,000; D and H, TEM at ×30,000.

The cell morphology of SFZP mutant *S.* Typhimurium during metal restriction was examined. SFZ and SFZP mutants grown in LB supplemented with 625 μM dipyridyl were sampled at 3 and 4 h and visualized by either differential interference contrast (DIC) or transmission electron microscopy (TEM). DIC images of cells from SFZ mutant cultures (Fig. 2A and C) appeared smooth, without surface defects, and TEM (Fig. 2B and D) revealed that the cell and outer membrane remained intact after 3 or 4 h of metal restriction. In contrast, DIC images of SFZP mutant cells at 3 h (Fig. 2E) showed blebbing of the cell surface, and TEM (Fig. 2F) revealed cytoplasmic extrusion. Cell blebbing was frequently located near the septum of dividing cells. After 4 h of growth in dipyridyl, the surface blebs of SFZP mutant cells had increased in size (Fig. 2G), with evident leakage of the intracellular contents (Fig. 2H). These results show that metal restriction of SFZ mutant *Salmonella* does not compromise the integrity of the cell envelope, provided that the Psp response is intact. In the absence of the Psp response, membrane integrity is compromised when essential metal uptake is restricted.

### The Rcs system maintains Δψ in metal-restricted *pspA* mutant *S.* Typhimurium.

**FIG 3:**
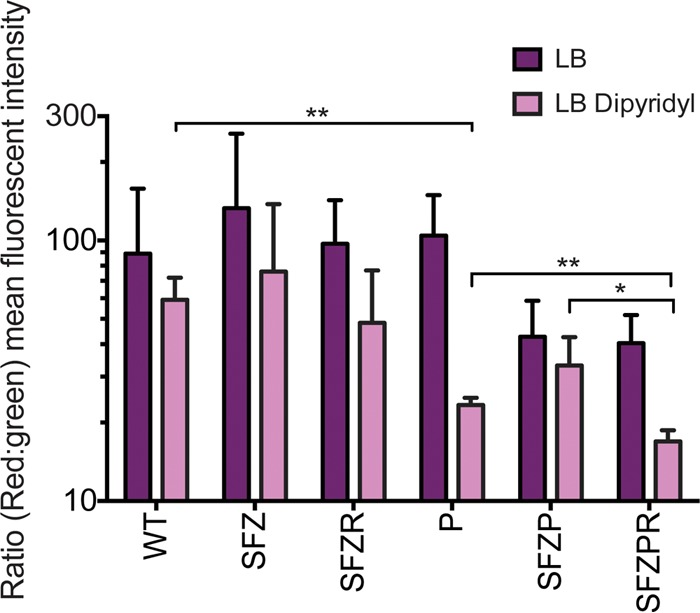
The Psp and Rcs responses maintain Δψ in metal transport-deficient mutants during growth in metal-limited medium. Δψ was measured by flow cytometry of aliquots from cultures incubated for 2 h in LB with 625 μM dipyridyl. Flow cytometry was performed with live bacterial cells following 15 min of incubation with the Δψ-sensitive dye DiOC_2_(3), which exhibits green fluorescence that shifts toward red fluorescence following Δψ-dependent intracellular aggregation. Data are expressed as the mean red-to-green emission ratio of a population of 2 × 10^4^ cells; a representative plot of three biological replicates is shown. Statistical significance was determined with an unpaired *t* test (*, *P* < 0.05; **, *P* < 0.01). Abbreviations: S, *sitA*; F, *feoB*; Z, *zupT*; P, *pspA*; R, *rcsA*.

The membrane abnormalities observed in SFZP mutant *S.* Typhimurium adjacent to the septum of dividing cells are similar to what has been previously observed in mutant strains lacking the Pal lipoprotein (47). Pal is part of the Tol-Pal complex that bridges the inner and outer membranes via protein-protein and protein-peptidoglycan interactions (48). The Tol-Pal complex is required for cell envelope integrity and is dependent on PMF (48, 49). Thus, the morphology of SFZP *S.* Typhimurium cells during metal deprivation suggests that PMF is compromised under these conditions. As the Psp response is known to maintain PMF under stress conditions and the Rcs system is strongly induced in metal-restricted SFZP mutant *S.* Typhimurium (Fig. 1A), we investigated whether the Rcs system helps to sustain the Δψ (membrane potential) component of PMF. WT and mutant *Salmonella* cultures were grown for 2 h in LB with or without 625 μM dipyridyl, and aliquots were removed and treated with DiOC_2_(3) dye for 15 min. DiOC_2_ (3) bound to the cell surface emits green fluorescence, whereas internalized DiOC_2_(3) aggregates and emits red fluorescence. As DiOC_2_(3) internalization is Δψ dependent, the red-to-green fluorescence ratio is a measurement of Δψ. Fluorescence was measured by flow cytometry, with the red-to-green fluorescence ratio interpreted as proportional to Δψ (Fig. 3). No difference in Δψ was observed between WT and mutant strains grown in LB under nonstress conditions or SFZ (*sit feo zup*) or SFZR (*sit feo zup rcsA*) mutants under metal-restricted conditions. In contrast, the Δψ of a *pspA* mutant was significantly lower than that of the WT during metal restriction, demonstrating that PspA is required to maintain Δψ under stress conditions, in agreement with the earlier observation that the Psp response facilitates metal transport (40). An SFZPR (*sit feo zup psp rcsA*) mutant under metal-restricted conditions had the lowest Δψ, indicating that the Rcs system can sustain the Δψ during stress when the Psp response is absent. Collectively, these observations suggest that the Psp response has a primary role in the conservation of Δψ and that the Rcs system can partially compensate for the absence of the Psp response to maintain Δψ.

### Construction of *S.* Typhimurium *yjb* and colanic acid capsule operon mutants.

The 19-gene colanic acid capsule operon and the *yjbEFGH* operon are regulated by the RcsA-RcsB heterodimer (19). To determine the contributions of colanic acid and the *yjbEFGH-*encoded EPS to Rcs-related phenotypes, deletion mutations of each operon were constructed. Recently, Ranjit and Young reported that a mutation in the colanic acid capsule operon downstream of the initiating glycosylase WcaJ can result in the accumulation of toxic pathway intermediates (50). This is of concern because prior investigations have used the disruption of single pathway genes or genes downstream of WcaJ to infer the biological role of colanic acid (50). Complete operon deletions were constructed to avoid this problem.

**FIG 4:**
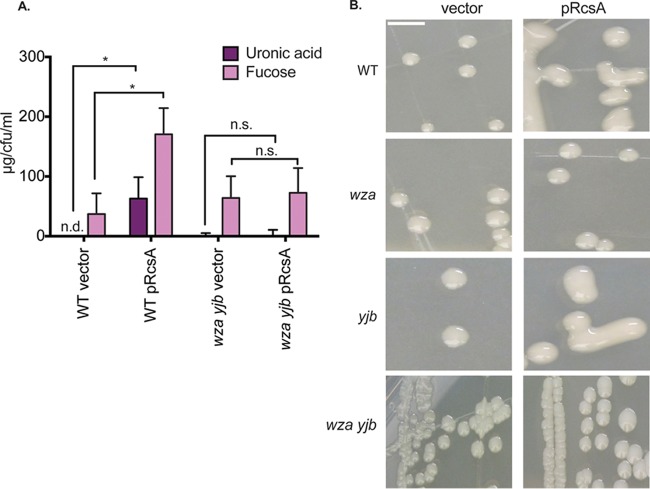
Mutations in the *wza* colanic acid and *yjbEFGH* operons eliminate EPS production. Expression of the colanic acid and *yjbEFGH* regulons is dependent on the RcsCDB phosphorelay. EPS production was induced by RcsA expressed in *trans* on a pBR322 replicon. (A) Purified EPS was subjected to a spectrophotometric assay for fucose and uronic acid. Values represent the mean of three biological replicates ± the standard deviation, and significance was determined with an unpaired *t* test (*, *P* < 0.05; n.s., not significant; n.d., not detected). (B) Representative images of colonies on LB agar plates formed by the WT strain or EPS-deficient mutants. Each strain contains either the pRcsA expression plasmid or the vector control. Colonies were allowed to grow for 3 days at 25°C. Scale bar, 5 mm.

Most *E. coli* and *S. enterica* strains are able to produce the colanic acid capsule (51, 52). Complete deletions of the colanic acid and *yjbEFGH* operons were constructed in *S. enterica* by λ-Red-mediated recombination (53, 54). The mutations were verified by molecular (see Materials and Methods) and functional assays involving the measurement of capsular carbohydrates (Fig. 4A). The colanic acid capsule is composed of glucose, galactose, fucose, and glucuronic acid (25, 26). The structure of the Yjb EPS has not been precisely determined, but it is known to contain a uronic acid component and lack fucose (18). WT cells overexpressing RcsA in *trans* showed a significant increase in both uronic acid and fucose relative to a vector control. The increase in uronic acid and fucose was eliminated by *wza* and *yjb* mutations. Colanic acid capsule overproduction results in mucoid colonies (25). Colony morphology further confirmed the lack of colanic acid production by a *wza yjb* mutant (Fig. 4B). Additional confirmation that neither the colanic acid capsule nor the Yjb EPS was being produced was provided by the failure to observe an increase in uronic acid (colanic acid and Yjb) or fucose (colanic acid) or the generation of mucoid colonies (colanic acid) in *wza yjb* mutants overexpressing RcsA.

### EPS deficiency enhances the sensitivity of *pspA* mutant *S.* Typhimurium to CAMPs.

**FIG 5:**
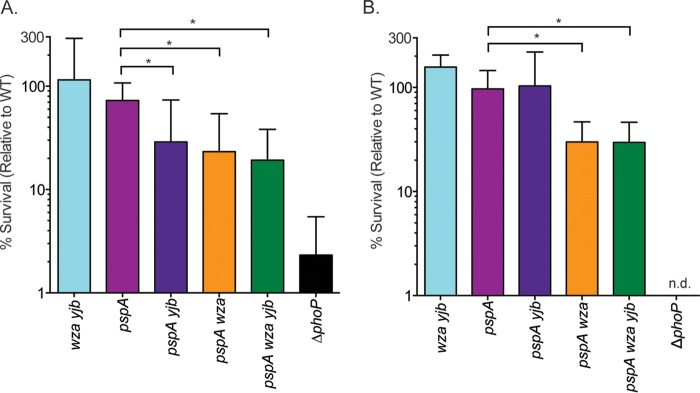
*S.* Typhimurium *pspA* mutants deficient in EPS production have increased sensitivity to antimicrobial peptides. (A) Sensitivity to the cationic BPI-P2 peptide (8 µg ml^−1^) was measured by enumerating CFU after 45 min of treatment at 37°C. Input CFU counts were calculated at time zero by plating untreated samples. Percent survival was calculated by dividing the CFU count at 1 h by the input CFU count and normalizing the result to the WT count. (B) Sensitivity to PMB (1 µg ml^−1^) was determined by enumerating CFU after 1 h of treatment at 37°C. Percent survival was calculated as described for P2. The mean percent survival ± the standard deviation of a minimum of four biological replicates is shown. Significance was determined by paired *t* test (*, *P* < 0.05; n.d., not detected).

CAMPs are amphipathic molecules that disrupt bacterial membranes and dissipate the PMF. The cationic P2 peptide derived from bactericidal/permeability-increasing (BPI) protein, BPI-P2, permeabilizes the bacterial outer membrane and disrupts energy-dependent processes (55). An *rpoE pspA* mutant *S.* Typhimurium strain has been previously shown to exhibit enhanced sensitivity to BPI-P2 (38). To determine whether the colanic acid and Yjb EPS protect cells from PMF-dissipating agents, we tested the susceptibility of WT and mutant strains to BPI-P2. The *pspA* and *wza yjb* mutants survived as well as the WT following exposure to 8 μg ml^−1^ BPI-P2 for 45 min at 37°C (Fig. 5A). However, *pspA yjb*, *pspA wza*, and *pspA wza yjb* mutants were significantly more sensitive to BPI-P2 than an isogenic *pspA* mutant strain.

The Rcs system has been previously implicated in sensitivity to the CAMP polymyxin B (PMB), independent of the colanic acid capsule (27). The sensitivity of WT and mutant strains to PMB was tested to determine if the capsules are required for PMB resistance in a *pspA* mutant background. Exposure to 1 μg ml^−1^ PMB for 1 h at 37°C did not affect the survival of a *pspA*, *wza yjb*, or *pspA yjb* mutant strain (Fig. 5B). However, *pspA wza* and *pspA wza yjb* mutants were significantly more sensitive to PMB than a *pspA* mutant, indicating that colanic acid but not the Yjb EPS promotes cell survival following PMB-mediated membrane damage in a *pspA* mutant. As the antimicrobial activity of CAMPs is dependent in part on PMF disruption, these observations are consistent with a role for colanic acid in PMF maintenance.

### The Psp and Rcs stress responses maintain Δψ in stationary phase.

**FIG 6:**
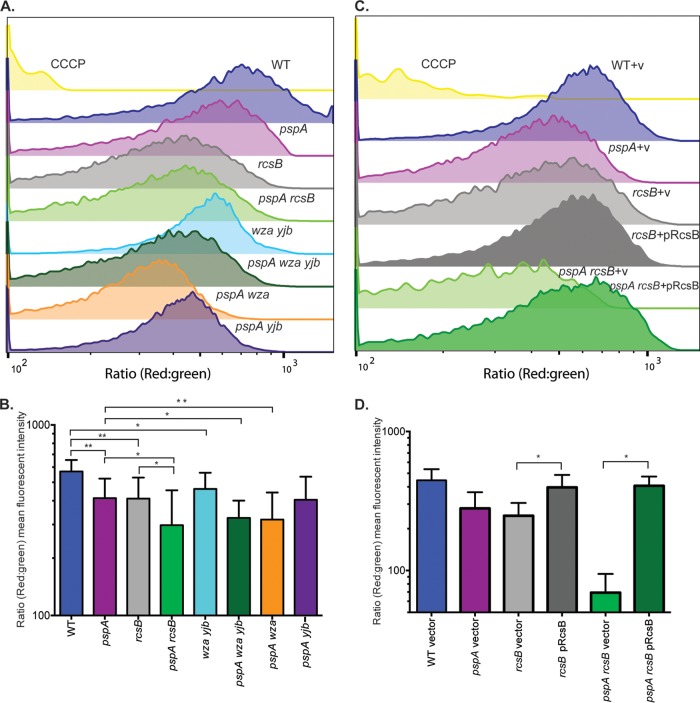
Rcs-regulated colanic acid capsule production maintains Δψ in stationary phase. *Salmonella* cultures were grown to stationary phase, and Δψ was measured by flow cytometry with DiOC_2_(3) as described in the legend to Fig. 3. Histograms show the red-to-green emission ratio as a measurement of Δψ distribution in a population of 2 × 10^4^ cells. The MFI of each histogram was determined from four biological replicates. WT cells depolarized by CCCP were included as a control. (A) Representative histograms of WT and mutant cells. (B) Replicate MFIs of the strains represented in panel A. (C) Representative histogram showing mutants complemented with pRcsB. (D) Replicate MFIs of strains represented in panel C. Significance was determined by paired *t* test (*, *P* < 0.05; **, *P* < 0.01).

The importance of the Psp response during stationary phase is well established. PspA is one of the most highly expressed proteins in stationary phase (1). Δψ and survival are both decreased during stationary phase in mutants lacking PspA (1, 38). The Δψ of WT and mutant bacteria was measured to determine whether the Rcs system, colanic acid capsule, and Yjb EPS contribute to the maintenance of this component of PMF in early stationary phase. Although our initial experiments focused on phenotypes dependent on RcsA, some residual capsule synthesis can be observed in *rcsA* mutant strains as a result of capsular operon activation by the RcsB homodimer (14). Therefore, Δψ was measured in *rcsB* mutants that are completely incapable of capsule production. Overnight cultures were diluted 1:1,000 in fresh LB and grown at 37°C with agitation to an OD_600_ of 1.5. Aliquots were taken, and the Δψ was measured by using DiOC_2_(3) and flow cytometry. The Δψ of individual cells (Fig. 6A and C) is depicted as histograms representing the distribution of red-to-green fluorescence ratios in populations of 2 × 10^4^ cells. The distribution of a WT population treated with the protonophore carbonyl cyanide *m*-chlorophenylhydrazone (CCCP) was included as a control, showing a left-shifted histogram with a lower red-to-green fluorescence ratio, indicating depolarization of the Δψ. The Δψ was measured in four biological replicate experiments, and the average mean fluorescence intensities (MFIs) were calculated from histograms for statistical analysis (Fig. 6B and D). The WT histogram (Fig. 6A) appears normally distributed, with an average MFI of 570 ± 85 (Fig. 6B). All mutants showed left-shifted distributions relative to the WT, with mean MFIs significantly different from that of the WT, confirming the requirement of PspA for the maintenance of Δψ during stationary phase and demonstrating a role for the Rcs response system in WT cells. Although the histogram of an *rcsB* mutant appears slightly left shifted in comparison to that of a *pspA* mutant, the mean MFIs are comparable (*pspA*, 413 ± 110; *rcsB*, 411 ± 118). The *pspA yjb* mutant histogram was only slightly left shifted compared to that of a *pspA* mutant, and the mean MFIs were not significantly different, indicating the Yjb EPS is not required for Δψ maintenance in stationary phase. The *pspA rcsB*, *pspA wza yjb*, and *pspA wza* mutant histograms were all left shifted relative to that of a *pspA* single mutant and slightly left shifted in comparison to that of an *rcsB* single mutant. Statistical analyses of the mean MFIs showed significantly lower red-to-green fluorescence ratios in *pspA rcsB*, *pspA wza yjb*, and *pspA wza* mutant strains than in *pspA* and *rcsB* single mutant strains. Together, these observations demonstrate that the Psp and Rcs stress responses contribute independently to the maintenance of Δψ in stationary phase and that colanic acid capsule production is specifically required. Expression of *rcsB* on a plasmid fully complemented an *rcsB* mutation and also restored Δψ in a *pspA* mutant strain (Fig. 6C and D).

### The Psp response and colanic acid capsule contribute to biofilm formation.

**FIG 7:**
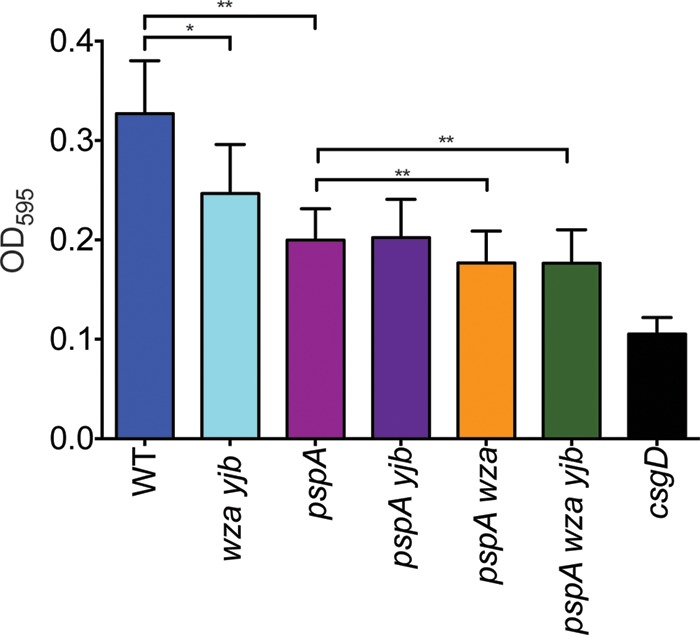
Biofilms formed by WT and mutant *Salmonella* strains. Strains were added to 96-well PVC microtiter plates containing LB and incubated without agitation at 25°C for 48 h. CV binding to biofilms was quantified by measuring absorbance at 595 nm. A *csgD* mutant lacking curli and cellulose was included as a biofilm negative control. Biofilm formation in five biological replicates was measured, and the mean values ± the standard deviation are shown. Statistical significance was determined by paired *t* test (*, *P* < 0.05; **, *P* < 0.01).

*Salmonella* can form biofilms on biotic and abiotic surfaces, including glass, plastic, gallstones, HEp-2 cells, and chicken intestinal epithelium (31, 56–58). The contribution of the colanic acid capsule to biofilm formation is well established (31, 32), and induction of the Psp response has been observed in biofilms (59). Therefore, we tested whether the inability to mount the Psp response and produce the colanic acid capsule impacts biofilm formation in microtiter plates. For biofilm formation, overnight cultures were adjusted to an OD_600_ of 1.0, diluted 1:100 in LB, and then added to microtiter plate wells and grown statically for 48 h at 25°C. Biofilms were quantified by the amount of crystal violet (CV) bound to EPS as measured by absorbance at 595 nm. No defect in growth was observed in any of the strains under these assay conditions, as determined by OD_600_ measurement (data not shown). The *pspA* mutant formed significantly less biofilm than WT cells (Fig. 7), demonstrating the importance of the Psp response for *Salmonella* biofilm formation. Biofilms formed by *pspA* and *pspA yjb* mutants were similar, whereas *pspA wza* and *pspA wza yjb* mutants formed significantly less biofilm than a *pspA* mutant. These observations indicate that the Psp response and the colanic acid capsule contribute to *S.* Typhimurium biofilm formation, whereas the Yjb EPS does not, as previously observed (8). Decreased biofilm formation by the *wza yjb* mutant is also likely to result from absence of the colanic acid capsule, further confirming that this EPS is essential for *Salmonella* biofilm formation. The red, dry, and rough (RDAR) colony morphotype is indicative of biofilm formation by *Salmonella* on agar plates containing the dyes Congo red and Coomassie blue (57). WT RDAR colonies are not formed by *pspA*, *wza yjb*, or *pspA wza yjb* mutants (see Fig. S2), providing additional evidence that the Psp response and the colanic acid capsule support biofilm development.

10.1128/mBio.00808-17.3 FIG S2  RDAR colony formation requires the Psp response and EPS production. *S.* Typhimurium strains were grown overnight in LB broth and plated on LB agar containing the dyes Congo red and Coomassie blue without salt. Colonies were grown for 7 days at 25°C. The *csgD* mutant is unable to form RDAR colonies and is included as a negative control. Images shown are representative examples. Download FIG S2, PDF file, 0.7 MB. Copyright © 2017 Pando et al. 2017 Pando et al. This content is distributed under the terms of the Creative Commons Attribution 4.0 International license.

### Colanic acid-deficient mutants are more susceptible to ampicillin.

**FIG 8:**
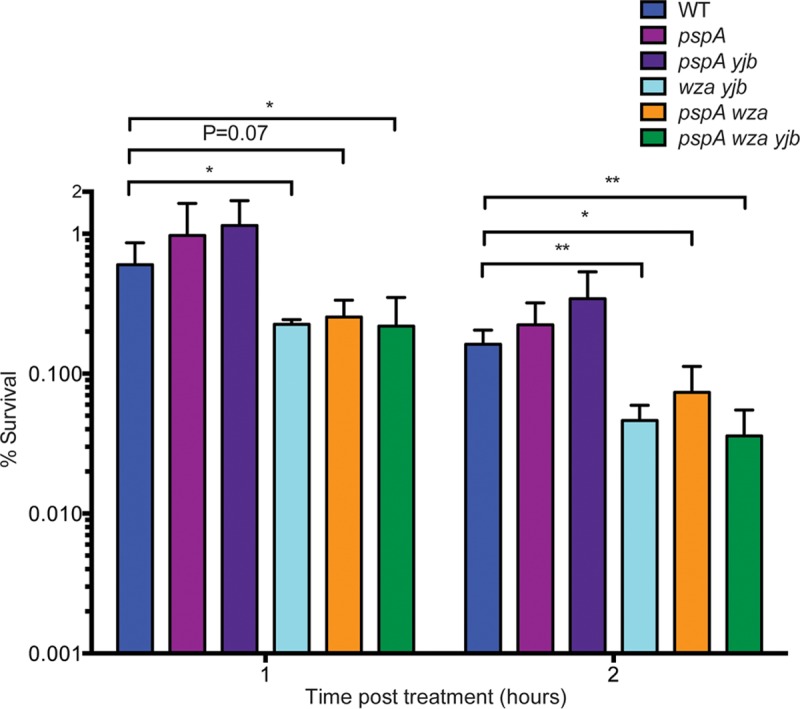
*S.* Typhimurium colanic acid capsule mutants show increased sensitivity to ampicillin. Strains were grown for 3 h to logarithmic phase, at which point 200 μg ml^−1^ ampicillin was added to culture tubes. Cultures were allowed to continue growth in the presence of antibiotic, samples were taken and plated at the time points indicated, and CFU were enumerated after 24 h. Susceptibility was determined by dividing the CFU count at the posttreatment times indicated by the CFU count of cultures immediately before antibiotic addition. The average survival ± the standard deviation of four biological replicates are shown. Significance was determined with a paired *t* test (*, *P* < 0.05; **, *P* < 0.01).

Beta-lactam antibiotics induce the colanic acid capsule and Yjb EPS, as well as the Psp operon (6, 9, 60). Sensitivity to ampicillin was measured to determine if the Psp response, colanic acid, or the Yjb EPS is protective against this clinically relevant antibiotic. Overnight cultures were used to inoculate fresh LB, and strains were grown to logarithmic phase before the addition of 200 μg ml^−1^ ampicillin and determination of survival by dilution, plating, and enumeration of CFU. A *pspA* mutation did not affect *Salmonella* sensitivity to ampicillin, nor did the introduction of a *yjb* mutation into a *pspA* mutant background (Fig. 8), at any time point measured. The *wza yjb*, *pspA wza*, and *pspA wza yjb* mutant strains, which lack the colanic acid capsular operon, all exhibited impaired survival following ampicillin treatment. Therefore, colanic acid supports *Salmonella* survival following ampicillin exposure.

## DISCUSSION

Although the extracytoplasmic stress responses of enteric bacteria react to different signals and control largely nonoverlapping sets of genes, there is substantial evidence that these responses can act in an integrated fashion. For example, the stress response controlled by the alternative sigma factor σ^E^ is expressed in response to the presence of misfolded outer membrane proteins in the periplasm and preserves cell envelope integrity (61). Abrogation of this response by the creation of an *rpoE* null mutation in *Salmonella* results in compensatory expression of the Cpx and Psp responses (37, 38). The CpxAR TCS senses the accumulation of misfolded proteins in the periplasm and responds by inducing protein-folding and -degrading factors (61) that have some functional overlap with the σ^E^ regulon (62). CpxR also cooperates with the BaeSR TCS in the regulation of certain genes (61) that respond to drug-induced envelope damage by activating the expression of efflux pumps and ameliorating oxidative stress (61, 63). Integration of the extracytoplasmic stress responses allows *Salmonella* to respond to a diverse array of environmental signals that threaten cell envelope integrity (39). Here, we describe the compensatory role of the *Salmonella* Rcs stress response system and colanic acid capsule production in the absence of the Psp response and the role of colanic acid in preserving PMF.

The Psp response was originally described as a system that preserves PMF in response to cell envelope disruption by filamentous bacteriophages (1). Our laboratory subsequently demonstrated that the essential role of the Psp response in *Salmonella* virulence is to support energy-dependent metal importation in the host environment (40). Unexpectedly, we observed that metal deprivation of a *Salmonella* strain lacking the Sit, Feo, and ZupT (SFZ mutant) metal transport systems caused the cells to lose viability if the Psp system was also inactivated (SFZP mutant) (40) (Fig. 1B). In the present study, we demonstrate that the loss of viability of an SFZP mutant is accompanied by loss of cell envelope integrity (Fig. 2). We hypothesize that metal depletion of an SFZP mutant impairs electron transport and results in energy depletion with a heightened dependency on the phage shock response to maintain PMF. The membrane instability observed in an SFZP mutant may result from disruption of the PMF-dependent formation of the cell envelope-stabilizing Tol-Pal complex (47), resulting in loss of viability.

A transcriptomic analysis of an SFZP mutant under metal-deprived conditions revealed expression of the Rcs system (see Table S1), which was confirmed by qPCR (Fig. 1A). Expression of the RcsA regulator from a plasmid is able to restore growth to the SFZP mutant in metal-deprived medium (Fig. 1B), indicating that the Rcs system is playing a compensatory role in the absence of PspA. *Salmonella* SFZP mutants continue to exhibit envelope structural defects (Fig. 2E to H) despite Rcs induction, indicating that the endogenous expression of Rcs is insufficient to completely compensate for the loss of the Psp response under these environmental conditions.

In view of the established role of the Psp response in PMF maintenance during envelope stress (1), we investigated whether the Rcs system also affects the Δψ component of PMF. We observed that metal deprivation of *Salmonella* results in depolarization of the Δψ, which is sustained by the Psp response (Fig. 3). Under these conditions, the Δψ of an SFZPR mutant lacking both the Psp and Rcs stress responses is significantly lower than that of mutants lacking only the Psp response. This suggests that the Rcs system helps to preserve PMF in the absence of the Psp response. As an *S.* Typhimurium *pspA* mutant was previously found to have attenuated virulence for mice expressing the metal transporter Nramp1 (40), we determined whether a *pspA rcsB* mutant was less virulent than a *pspA* mutant during *S.* Typhimurium infection of Nramp1-expressing C3H/OuJ Nramp^+^ mice. However, a competitive-infection experiment showed no effect of an *rcsB* mutation on virulence in this model (see Fig. S3). Further attenuation of the virulence of a *pspA rcsB* mutant was not observed, possibly because the effects of a *pspA* mutation on virulence are sufficiently marked that further attenuation could not be detected. Other investigators have found that *rcsB* mutants can be outcompeted by WT *S.* Typhimurium after 3 weeks of competitive infection of 129SvC6 mice (27).

10.1128/mBio.00808-17.4 FIG S3  *Salmonella* virulence requires the Psp but not the Rcs stress response. Six-week-old C3H/OuJ Nramp^+^ mice were infected intraperitoneally with an ~1:1 mixture of mutant and WT bacteria. On day 5 postinfection, coinfected mice (*n* = 10) were euthanized and the CFU in their livers and spleens were enumerated. A value of <1 indicates a competitive advantage of the WT over the mutant. Bars represent the median values. Each symbol represents the result for one mouse. Statistical significance was determined with a Mann-Whitney test (*, *P* < 0.05; **, *P* < 0.01; n.s., not significant). Download FIG S3, PDF file, 0.3 MB. Copyright © 2017 Pando et al. 2017 Pando et al. This content is distributed under the terms of the Creative Commons Attribution 4.0 International license.

Other conditions that stimulate colanic acid production are also known to perturb membrane energetics. For example, IgA monoclonal antibody Sal4 impairs membrane integrity, transiently reduces PMF (64), and induces colanic acid synthesis (65). Low concentrations of the CAMP PMB permeabilize the cell membrane and disrupt respiration, and higher PMB concentrations result in Δψ depolarization (66). PMB also induces colanic acid synthesis (67), and we observed that absence of the colanic acid capsule renders *pspA* mutant *Salmonella* more susceptible to this antimicrobial agent (Fig. 5B).

Although elimination of the Psp response by itself did not affect the survival of cells exposed to the CAMP PMB or BPI-P2, elimination of both the Psp response and colanic acid synthesis enhanced susceptibility to both peptides (Fig. 5). Deletion of the *yjbEFGH* operon did not increase the susceptibility of a *pspA* mutant to PMB but enhanced its sensitivity to the BPI-P2 antimicrobial peptide (67), suggesting that the Yjb EPS subserves a similar function.

Both the Psp (1) and RcsB (68) responses are induced during stationary phase. Stationary-phase cultures of *pspA* or *rcsB* mutant *Salmonella* exhibited significantly lower Δψ than the WT (Fig. 6A and B), and the Δψ of a *pspA rcsB* double mutant was even lower, demonstrating that both the Psp and Rcs stress responses maintain Δψ in stationary phase. Measurement of Δψ in *pspA* mutants lacking either the *wza* or *yjb* operon indicated that colanic acid, but not the Yjb EPS, is essential for Δψ maintenance during stationary phase. With the construction of an *rcsB* mutation, which completely abolishes expression of the Rcs regulon (14), we also found that the Rcs system is required for Δψ preservation, even in the presence of the Psp response (Fig. 5), and can be restored by the expression of RcsB in *trans* (Fig. 6B). We did not observe a decrease in Δψ in a *wza yjb rcsB* mutant beyond what is observed in the *rcsB* mutant, suggesting that no additional RcsB-regulated factors are required for PMF maintenance (see Fig. S4).

10.1128/mBio.00808-17.5 FIG S4  RcsB-regulated colanic acid capsule maintains stationary-phase Δψ. *Salmonella* cultures were grown to early stationary phase, and Δψ was measured as described in the legend to Fig. 6. (A) Representative histograms for WT and mutant cells. (B) Replicate MFIs from four biological replicates of the strains represented in panel A. Statistical significance was determined with a paired *t* test (*, *P* < 0.05). Download FIG S4, PDF file, 0.4 MB. Copyright © 2017 Pando et al. 2017 Pando et al. This content is distributed under the terms of the Creative Commons Attribution 4.0 International license.

Colanic acid is highly expressed in *Salmonella* biofilms, most likely to address membrane bioenergetic requirements during slow growth and nutrient limitation (30, 59, 69). Decreased biofilm formation by a *pspA* mutant (Fig. 7) suggests that PMF is reduced in biofilms, and *Salmonella* mutants lacking both *pspA* and the colanic acid capsule formed even less biofilm, suggesting that, in addition to its proposed structural role, colanic acid may function to maintain membrane energetics in biofilms as well. Bacteria in biofilms are notable for their resistance to killing by antibiotics (70), and we observed that colanic acid capsule biosynthesis contributes to resistance to ampicillin (Fig. 8), an antibiotic used to treat *Salmonella* infections. Thus, the colanic acid capsule contributes to the antibiotic tolerance of *Salmonella* in biofilms.

The strongly negative charge of colanic acid (26) is likely to account for its ability to maintain Δψ under stress conditions. A negative charge adjacent to the bacterial cell surface requires protons as counterions. A local increase in the proton concentration at the cell surface can enhance both the surface potential and ΔpH, which has been shown to increase ATP generation in *E. coli* (71).

The *yjbEFHG* operon appears to be associated with the production of a distinct type of EPS, but its structure and cell association have not been defined (8). We therefore cannot say why it is unable to maintain PMF in the absence of colanic acid. In the future, the analysis of other types of capsule whose structures and charge are characterized may provide further insight into the mechanism of PMF maintenance by colanic acid.

Our observations corroborate Model’s original hypothesis that the Psp response conserves PMF under stress conditions and provides evidence that the Rcs system and specifically colanic acid also contribute to this function. This demonstrates a novel physiological role for the colanic acid capsule that may provide a unifying mechanism to account for its diverse contributions to stress resistance in enteric bacteria.

## MATERIALS AND METHODS

For additional information regarding our materials and methods, see Text S1, and for information about the strains, plasmids, and primers used, see Table S2.

10.1128/mBio.00808-17.1 TEXT S1  Supplemental materials and methods. Download TEXT S1, PDF file, 0.05 MB. Copyright © 2017 Pando et al. 2017 Pando et al. This content is distributed under the terms of the Creative Commons Attribution 4.0 International license.

10.1128/mBio.00808-17.7 TABLE S2  Strains, plasmids, and primers used in this study. Download TABLE S2, PDF file, 0.1 MB. Copyright © 2017 Pando et al. 2017 Pando et al. This content is distributed under the terms of the Creative Commons Attribution 4.0 International license.

### Bacterial growth conditions.

All strains were routinely cultured in LB with shaking at 250 rpm at 37°C unless otherwise stated. Antibiotics were used at the following concentrations, as indicated: ampicillin, 100 μg ml^−1^; kanamycin, 50 μg ml^−1^; chloramphenicol, 20 μg ml^−1^; tetracycline, 25 μg ml^−1^.

### Strain and plasmid construction.

Mutant strains were constructed with the λ-Red recombinase system (54). The *wza* colanic acid capsule mutant was constructed by the λ-Red *tetRA* replacement method (53). All mutations were verified by PCR with gene-specific primers and transduced into a clean 14028s background with bacteriophage P22. To generate plasmid JP102, plasmid pATC118 (17) was digested with EcoRI and HindIII to generate an 860-bp DNA fragment containing the Δ37 *rcsA* complementing fragment. The 860-bp fragment was then cloned into pJK392 at the EcoRI and HindIII sites and ligated with T4 DNA ligase (New England Biolabs, Ipswich, MA). To generate plasmid JP103, primers JPP249/250 were used to PCR amplify the *rcsB* promoter and coding region (68). Primers were designed to include the −35 and −10 elements of P_*rcsB*_, which are located within the *rcsD* coding region (68). The *rcsB* gene was cloned into stable low-cloning vector pRB3-273C (72) at the SmaI site and verified by sequencing.

### Flow cytometry.

Overnight cultures were diluted 1:1,000 in fresh LB containing the metal chelator 2′2′-dipyridyl (Sigma-Aldrich) at 625 μM in a volume-to-flask ratio of 9:25. After 2 h of growth, approximately 1 × 10^6^ CFU were added to a 5-ml flow cytometry tube containing 1 ml of permeabilization buffer (10 mM Tris [pH 7.5], 1 mM EDTA) and 30 μM DiOC_2_(3) (Sigma-Aldrich) and incubated in the dark for 15 min at room temperature. A total of 2 × 10^4^ cells were assayed with an LSRII flow cytometer with a 488-nm excitation wavelength. Green emission was detected through a 505-nm long-pass filter with a 530- to 30-nm bandpass filter, and red emission was detected through a 600-nm long-pass filter with a 610- to 20-nm bandpass filter. Gates for bacterial populations were based on the WT population by using forward versus side scatter and red versus green emission. For measurements of stationary-phase cultures, overnight cultures were diluted 1:1,000 in fresh LB in a volume-to-flask ratio of 1:5, grown to an OD_600_ of ~1.5, and then assayed by flow cytometry as already described. Flow cytometry data were processed with FlowJo v 10.0.7 software (TreeStar, Inc.) and analyzed by using the red-to-green fluorescence ratio as previously described (73). Flow cytometry was performed at the University of Washington Pathology Flow Cytometry Core Facility.

### Capsule purification and quantification.

An overnight culture was diluted 1:1,000 in 50 ml of fresh LB with ampicillin and grown to an OD_600_ of ~2.0. One milliliter of the 50-ml culture was used to enumerate CFU by dilution and plating on LB agar, and 25 ml was pelleted, resuspended in an equal volume of phosphate-buffered saline (PBS), and then boiled for 15 min to inactivate EPS-degrading enzymes and completely release EPS from the cell surface. The boiled sample was allowed to cool to room temperature and then centrifuged at 25,400 × *g* for 30 min at 4°C, and the supernatant was combined with 3 volumes of 70% ethanol and incubated overnight at 4°C. Following overnight incubation, the sample was centrifuged at 25,400 × *g* for 30 min at 4°C and the resulting pellet was resuspended in 1 ml of sterile water and dialyzed against distilled water for 48 h. The final sample was stored at 4°C until quantification. Total fucose and uronic acid contents were quantified in accordance with established protocols (74, 75). Total sugar contents were normalized to CFU counts and expressed in micrograms per CFU per milliliter.

### Susceptibility assays.

Measurement of PMB sensitivity was performed in glass culture tubes as previously described (76). PMB stock was made in a glass tube, stored at 4°C, and used for no longer than 1 week.

Synthesis of the BPI-P2 peptide was previously described (38). P2 sensitivity was determined by a previously developed method (77). Briefly, cultures were grown in Trypticase soy broth (TSB), diluted 1:100 in fresh TSB, and then grown to an OD_600_ of ~1.0. A total of 10^6^ bacteria ml^−1^ were treated with 8 μg ml^−1^ of BPI-P2 peptide, and cells were kept stationary at 37°C for 45 min. Input CFU counts were determined at time zero by plating unexposed samples on LB agar and counting colonies after 24 h at 37°C. Percent survival was determined by dividing the CFU count obtained after antimicrobial exposure by the input CFU count and normalizing the result to WT percent survival.

Susceptibility to 200 and 100 μg ml^−1^ ampicillin was determined as previously described (60). Briefly, overnight cultures were diluted 1:1,000 in fresh LB and grown for 3 h before plating to enumerate CFU before the addition of ampicillin and at the time points indicated following antibiotic treatment. Percent survival was calculated by dividing the CFU count obtained after ampicillin exposure by the unexposed input CFU count.

Growth kinetics in the presence of dipyridyl were performed as previously described (40), with a Bioscreen C Microbiology microplate reader (Growth Curves USA).

### Microscopy.

To prepare cells for microscopy, overnight cultures were diluted 1:1,000 in 1 liter of fresh LB with the metal chelator 2′2′-dipyridyl (Sigma-Aldrich) at 625 μM in a volume-to-flask ratio of 9:25. Cultures were grown with shaking at 37°C, and aliquots taken at the time points indicated, pelleted, and kept on ice. For DIC microscopy, pelleted cells were resuspended in 0.85% NaCl and 2 μl was immobilized on an agarose pad and imaged with a Nikon Eclipse TE200 inverted microscope. For TEM, cells were pelleted, washed two times with PBS, and resuspended in 1 ml of 0.5× Karnovsky fixative. TEM imaging was performed at the University of Washington Electron Microscopy Center.

### RNA preparation, cDNA synthesis, and qPCR.

Overnight cultures were diluted 1:1,000 in 1 liter of fresh LB with the metal chelator 2′2′-dipyridyl (Sigma-Aldrich) at 625 μM in a volume-to-flask ratio of 9:25, and 200 ml of cells was pelleted after 2 h of growth. The pellet was resuspended in 2.5 ml of Trizol reagent. Contaminating DNA was removed by a 1-h DNase (Fermentas) treatment. Following the DNase treatment step, the RNA was further purified by the acid-phenol method and stored at −80°C. RNA purity was determined on a 2% agarose gel and with a NanoDrop spectrophotometer. The Qiagen QuantiTect reverse transcription kit was used to synthesize cDNA with 500 ng of RNA as the input. qPCR was performed with the SYBR green kit (Qiagen, Valencia, CA) and the CFX96 real-time system (Bio-Rad, Hercules, CA) with *rpoD* as an internal control.

### CV-based biofilm assays.

Overnight cultures were brought to an OD_600_ of ~1.0 with fresh LB. Adjusted cultures were then diluted 1:100 in fresh LB in a 96-well polystyrene microtiter plate. Plates were sealed with Parafilm and incubated at 25°C for 48 h. The OD_600_ was measured to determine growth, and then culture supernatants were decanted, and unbound bacteria were removed by washing with PBS (pH 7.4). Remaining cells and cell-associated material were stained with 0.1% CV for 10 min. After staining, wells were washed twice with PBS and the dye was solubilized with an 80:20 (vol/vol) ethanol-acetone mixture. CV absorbance was quantified at 595 nm.
