# Supplementary material for: The Rcs-Regulated Colanic Acid Capsule Maintains Membrane Potential in Salmonella enterica serovar Typhimurium
Source: mBio. 2017 Jun 6;8(3):e00808-17. doi: 10.1128/mBio.00808-17 (PMC5461412; doi:10.1128/mBio.00808-17)
Supplement: TABLE S1 [file mbo003173339st1.pdf]

Table S1. Genes induced four-fold or greater during growth of a *Salmonella* SFZP mutant in LB with 625 μM dipyridyl.

SFZP = *sit feo zup psp*.

STM#	Gene Symbol[a]	Function[a]	Fold Change[b]
**Psp regulon**			
STM1690	*pspA*	phage shock protein A	23
STM1689	*pspB*	phage shock protein B	47.1
STM1688	*pspC*	phage shock protein C	71.3
STM1687	*pspD*	phage shock protein D	44.6
STM1686	*pspE*	phage shock protein E	26.1
STM4244	*pspG*	phage shock protein G	4.3
**RcsCDB regulon**			
STM2262	*eco*	ecotin	5.6
STM1982	*rcsA*	transcriptional regulator	4.5
STM1705	*osmB*	osmotically inducible lipoprotein B	5.3
STM1212	*ycfJ*	outer membrane lipoprotein	6.3
STM0414	*yajI*	outer membrane lipoprotein	4.3
Colanic acid capsule operon			
STM2118	*wza*	polysaccharide export protein	31.5
STM2117	*wzb*	protein-tyrosine-phosphatase	25.1
STM2116	*wzc*	colanic acid tyrosine-protein kinase	36
STM2115	*wcaA*	glycosyl transferase	28.2
STM2114	*wcaB*	colanic acid biosynthesis acetyltransferase	20.5
STM2113	*wcaC*	glycosyl transferase	20.2
STM2112	*wcaD*	colanic acid polymerase	14.7
STM2111	*wcaE*	glycosyl transferase	30
STM2110	*wcaF*	colanic acid acetyltransferase	44
STM2109	*gmd*	GDP-D-mannose dehydratase	78.9
STM2108	*wcaG*	GDP fucose synthetase	64.1
STM2107	*wcaH*	GDP-mannose mannosyl hydrolase	34.9
STM2106	*wcaI*	glycosyl transferase	31.3
STM2105.S	*manC*	mannose-1-phosphate guanylyltransferase	35.2
STM2104	*cpsG*	phosphomannomutase	26.7
STM2103	*wcaJ*	UDP-glucose lipid carrier transferase	13.3
STM2102	*wzxC*	colanic acid exporter	8.8
STM2101	*wcaK*	pyruvyl transferase	8.6
STM2100	*wcaL*	colanic acid biosynthesis glycosyl transferase	14.3
STM2099	*wcaM*	colanic acid biosynthesis protein	9.1
yjb operon			
STM4223	*yjbF*	outer membrane lipoprotein	16.2
STM4224	*yjbG*	periplasmic protein	13.9
STM4225	*yjbH*	outer membrane lipoprotein	11.2
**Others**			
STM4274	*yjcH*	inner membrane protein	8.2
STM3645	*yiaD*	outer membrane lipoprotein	7
STM3157	*yghA*	Oxidoreductase	4.9
STM1697		diguanylate cyclase/phosphodiesterase domain containing protein	5.6
STM1685	*ycjX*	ATPase	31
STM1684	*ycjF*	hypothetical protein	18.4

[a] Gene symbol and predicted protein function was obtained from the *S. enterica* serovar LT2 database on the BioCyc Database Collection website.
[b] Fold change denotes an increase in the SFZP mutant’s transcript levels relative to the SFZ mutant.
