# Supplementary material for: The Rcs-Regulated Colanic Acid Capsule Maintains Membrane Potential in Salmonella enterica serovar Typhimurium
Source: mBio. 2017 Jun 6;8(3):e00808-17. doi: 10.1128/mBio.00808-17 (PMC5461412; doi:10.1128/mBio.00808-17)
Supplement: FIG S1 [file mbo003173339sf1.pdf]

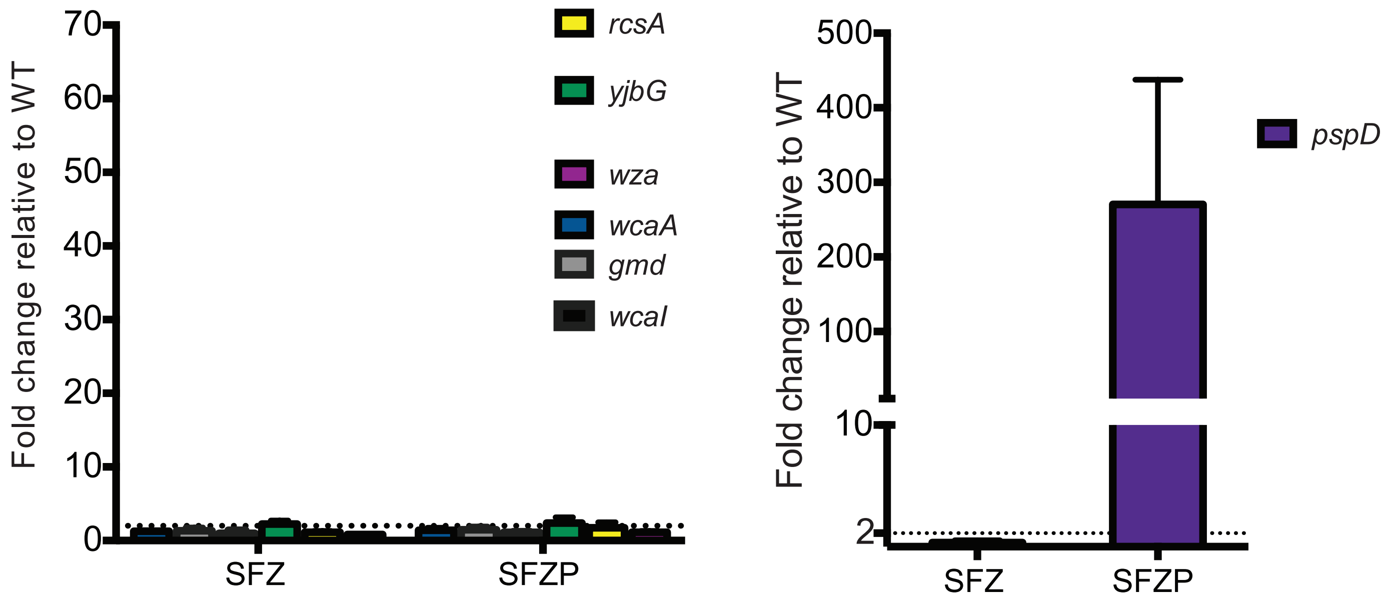

Fig. S1. Growth in LB does not induce *rcsA* or the colanic acid capsule operon. Quantitaitve PCR (qPCR) was performed with cDNA obtained from cultures grown in LB for 2 hrs. Absolute qPCR values were normalized to the bacterial housekeeping gene *rpoD* and expressed as the fold-change over wild-type. Mean qPCR values from 3 biological replicates ± SD are shown. The gene *pspD* is highly expressed in the SFZP mutant due to lack of the negative regulator PspA and is shown as a positive control.
