# Supplementary material for: The Rcs-Regulated Colanic Acid Capsule Maintains Membrane Potential in Salmonella enterica serovar Typhimurium
Source: mBio. 2017 Jun 6;8(3):e00808-17. doi: 10.1128/mBio.00808-17 (PMC5461412; doi:10.1128/mBio.00808-17)
Supplement: FIG S2 [file mbo003173339sf2.pdf]

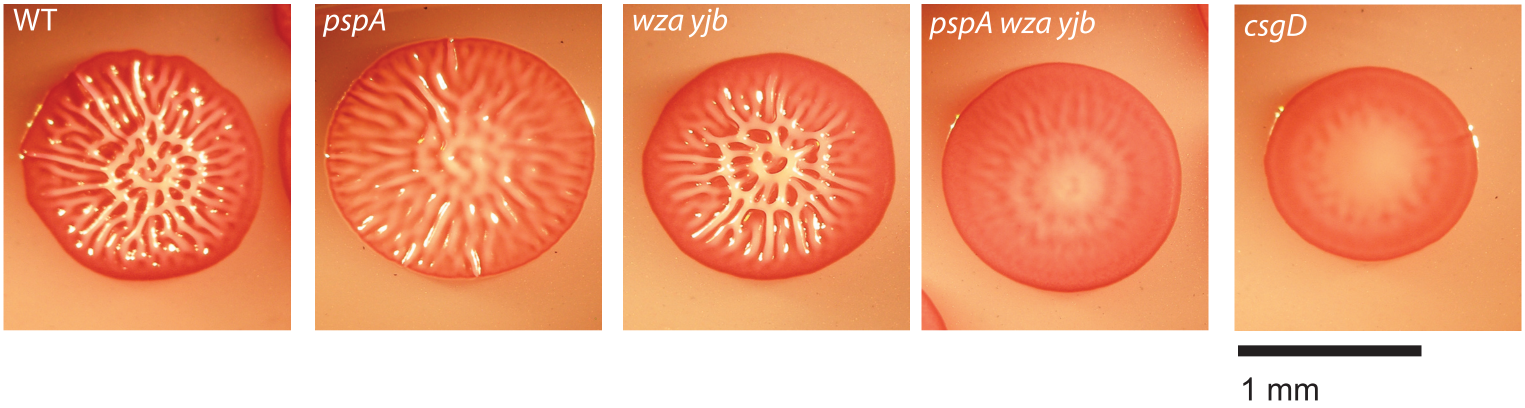

Fig. S2. RDAR colony formation requires the Psp response and exopolysaccharide production. *S*. Typhimurium strains were grown overnight in LB broth and plated onto LB agar containing the dyes Congo red and Coomassie blue without salt. Colonies were grown for 7 days at 25°C. The *csgD* mutant is unable to form RDAR colonies and is included as a negative control. Images shown are representative examples.
