# Supplementary material for: The Rcs-Regulated Colanic Acid Capsule Maintains Membrane Potential in Salmonella enterica serovar Typhimurium
Source: mBio. 2017 Jun 6;8(3):e00808-17. doi: 10.1128/mBio.00808-17 (PMC5461412; doi:10.1128/mBio.00808-17)
Supplement: FIG S3 [file mbo003173339sf3.pdf]

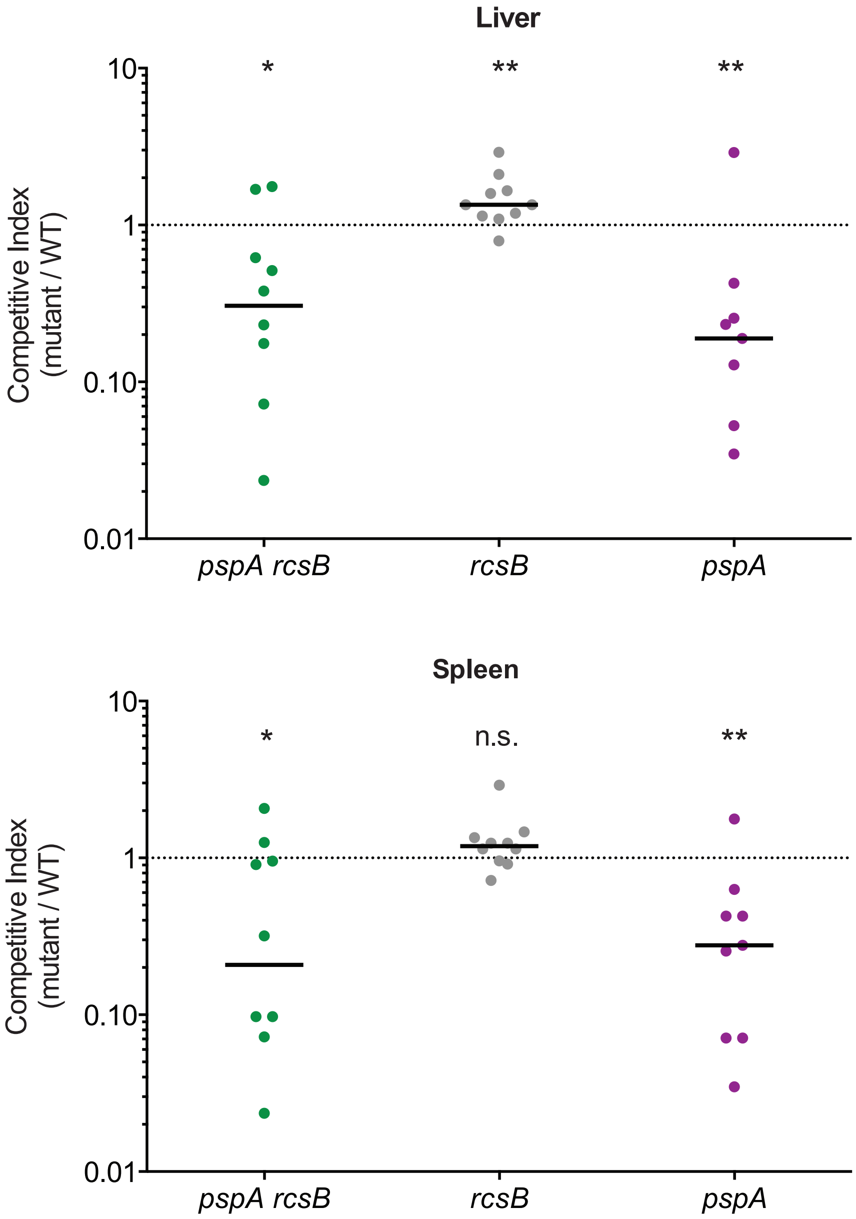

Fig.S3. The Psp response is required for systemic virulence but the Rcs system is not. Six week old C3H/OuJ Nramp[+] mice were infected i.p with an ~1:1 mixture of mutant and wild-type bacteria. On day 5 post-infection, co-infected mice (n =10) were euthanized and CFU in liver and spleen were enumerated. Values less than 1 indicate a competitive advantage of WT over mutant. Bars represent the median values. Each symbol represents the result for 1 mouse. Statistical significance was determined using a Mann-Whitney test (*P < 0.05; **P < 0.01; n.s., not significant).
