# Supplementary material for: The Rcs-Regulated Colanic Acid Capsule Maintains Membrane Potential in Salmonella enterica serovar Typhimurium
Source: mBio. 2017 Jun 6;8(3):e00808-17. doi: 10.1128/mBio.00808-17 (PMC5461412; doi:10.1128/mBio.00808-17)
Supplement: FIG S4 [file mbo003173339sf4.pdf]

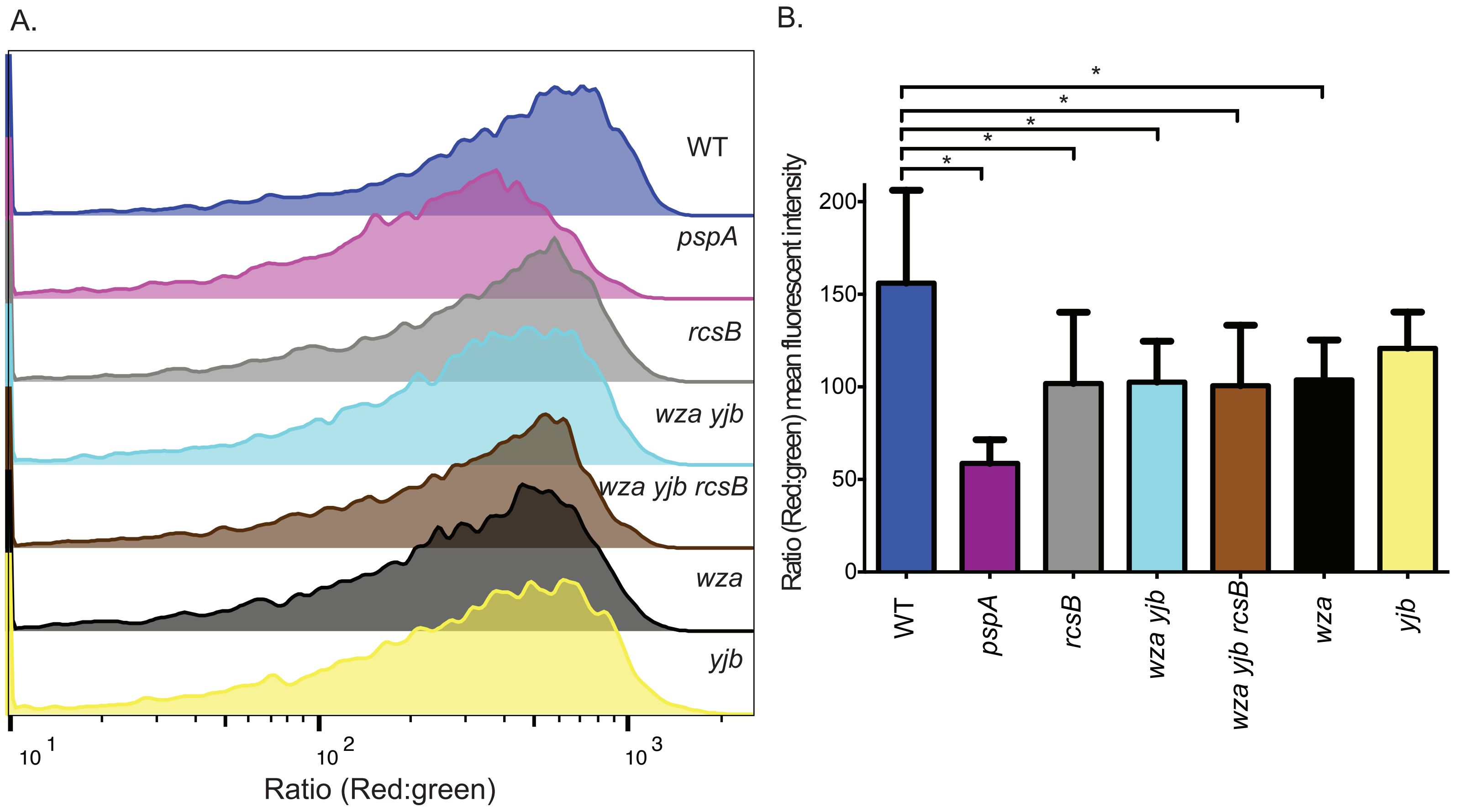

Fig. S4. RcsB regulated colanic acid capsule maintains stationary phase membrane potential. *Salmonella* cultures were grown to early stationary phase and membrane potential was measured as in Fig. 6. (A) Representative histograms for wild-type and mutant cells. (B) Replicate MFIs from 4 biological replicates for the strains represented in panel A. Statistical significance was determined using a paired t-test (*$P < 0.05$).
