# Supplementary material for: The Rcs-Regulated Colanic Acid Capsule Maintains Membrane Potential in Salmonella enterica serovar Typhimurium
Source: mBio. 2017 Jun 6;8(3):e00808-17. doi: 10.1128/mBio.00808-17 (PMC5461412; doi:10.1128/mBio.00808-17)
Supplement: TEXT S1 [file mbo003173339s1.pdf]

Materials and Methods

Mouse virulence assay

Mouse infections were performed similarly to those previously described, with modifications as noted (40). C3H/OuJ mice were purchased from Jax Laboratories (Bar Harbor Maine) and housed at the Modified Specific Pathogen Free facilities at the University of Washington animal facilities under protocol 3373-01. For competitive infections, a 50/50 mixture of strains WT and mutant was used to infect mice intraperitoneally. Five days post infection, liver and spleen where removed and homogenized in PBS by using an Ultra Turrax T25 basic mixer (IKA). Homogenates were serially diluted and plated on LB agar plates. One hundred colonies from each tissue type were picked onto LB agar containing tetracycline (20 µg ml^{-1}). Competitive indices (CI) were determined as the cfu of mutant divided by the CFU of WT, normalized to the input inoculum.

Detection of the RDAR colonial morphotype.

For data regarding the RDAR colonial morphotype, bacteria were grown as in (79) with modifications. Overnight cultures grown in LB broth were diluted to a concentration of ~100 CFU ml^{-1}. Aliquots of 100 µl were plated onto LB agar without salt and supplemented with 40 µg ml^{-1} Congo red and 20 µg ml^{-1} Coomassie blue. Plates were inverted and incubated at 25°C for 7 days (27).

Microarray

RNA was prepared from SFZ and SFZP cells as described in RNA preparation after 2 hours of growth in LB with 625 μM dipyridyl. Microarray was performed as described in (76).
