# Supplementary material for: The Rcs-Regulated Colanic Acid Capsule Maintains Membrane Potential in Salmonella enterica serovar Typhimurium
Source: mBio. 2017 Jun 6;8(3):e00808-17. doi: 10.1128/mBio.00808-17 (PMC5461412; doi:10.1128/mBio.00808-17)
Supplement: TABLE S2 [file mbo003173339st2.pdf]

Table S2.

**Strain, plasmid or primer**	**Genotype, relevant characteristics, or sequence**	**Source or reference**
Strains		
JP1	*Salmonella* Typhimurium 14028s wild type	ATCC
JP54	*sitA*::MudCm *feoB*::Tn10 Δ*zupT*:: FRT	(40)
JP55	*sitA*::MudCm *feoB*::Tn10 Δ*zupT*:: FRT Δ*pspA*:: FRT	(40)
JP2	Δ*pspA*:: FRT	(40)
JP52	*sitA*::MudCm *feoB*::Tn10 Δ*zupT*:: FRT Δ*rcsA*:: FRT *kan*FRT	this paper
JP53	*sitA*::MudCm *feoB*::Tn10 Δ*zupT*:: FRT Δ*pspA*:: FRT Δ*rcsA*:: FRT*kan*FRT	this paper
JP57	14028s/ pJP102	this paper

JP65	14028s/pJK392	this paper
JP260	*wza*::tetRA Δ*yjbE*::FRT*kan*FRT /pJP102	this paper
JP267	*wza*::tetRA Δ*yjbE*::FRT*kan*FRT /pJK392	this paper
JP312	Δ*pspA*:: FRT *wza*::tetRA	this paper
JP327	Δ*pspA*:: FRT Δ*yjbE*::FRT*kan*FRT	this paper
JP384	Δ*pspA*:: FRT *wza*::tetRA Δ*yjbE*::FRT*kan*FRT	this paper
JP391	Δ*rcsB*:: FRT*cat*FRT	this paper
JP400	Δ*pspA*:: FRT Δ*rcsB*:: FRT*cat*FRT	this paper
JP249	*wza*::tetRA Δ*yjbE*::FRT*kan*FRT	this paper
JP387	14028s/pRB3-273C	this paper
JP389	Δ*pspA*:: FRT /pRB3-273C	this paper

JP431	Δ*rcsB*:: FRT*cat*FRT /pRB3-273C	this paper
JP422	Δ*pspA*:: FRT Δ*rcsB*:: FRT*cat*FRT/ pRB3::14028sP$_{rcsB}$-*rcsB*	this paper
JP425	Δ*rcsB*:: FRT*cat*FRT/ pRB3::14028sP$_{rcsB}$-*rcsB*	this paper
JP412	Δ*pspA*:: FRT Δ*rcsB*:: FRT*cat*FRT/ pRB3-273C	this paper
CS051	14028s *phoP*102::Tn10dCm	(78)
SLN112	14028s *csgD::*FRT	this paper
JP245	*wza*::tetRA Δ*yjbE*::FRT*kan*FRT	this paper
JP447	Δ*yjbE*::FRT*kan*FRT	this paper
JP446	ΔrcsB::FRTcatFRT wza::tetRA Δ*yjbE*::FRT*kan*FRT	this paper
**Plasmids**		

pKD4	*bla* FRT *kan* FRT PS1 PS2 *ori6K*	(54)
pKD13	*bla* FRT *kan* FRT PS1 PS4 *ori6K*	(54)
pKD46	*bla araC*-P_{araB} γ β *exo* *oriR101* repA101(Ts)	(54)
pRB3-273C	*par* RK2 *bla* stable cloning vector	(72)
pJK392	Col E1 *bla*	
pRcsA	pJK392:: rcsA	this paper
JP103	pRB3::14028sP_{rcsB}-*rcsB*	this paper
**Primers**	**5' → 3'**	
JKP384-rscA-P1	GGGTATGCCATGTCAAC GATTATTATGGATTTGTG CAGTTGTGTAGGCTGGA GCTGCTTC	
JKP385-rscA-P2	ATGTCTCAGCGCATGTT AACAAAAATGCCGTTAG TGACGTCATATGAATATC CTCCTTAG	
JPP179-wza-tetR	CACAGGATAATGACTCT GCCAAAGTGATAAATAAT CAATGTTAAGACCCACTT TCACATT	

JPP180-wcaM-tetA	AACTTTTTCCCAGGAATT TTCGTAAAAATAGCGGT ACTAACTAAGCACTTGTC TCCTG	
JPP245-rcsB-P1	TACGTCAAAAGCTTGCT GTAGCAAGGTAGCCCAA TACATG GTG TAG GCT GGA GCT GCT TC	
JPP246-rcsB-P2	CCATCAGGCTGGGTAAC ATAAAAGCGATTTATTCT TTGTCCAT ATG AAT ATC CTC CTT AG	
JPP68-yjbE-P1	GGCACTGCGCTTATCCG GTC	
JPP69-yjbE-P2	CGATAAACTCAAGACGT GAG	
JPP42-*rpoD*-F	GTGAAATGGGCACTGTT GAACTG	

JPP43-*rpoD*-R	TTCCAGCAGATAGGTAA TGGCTTC	
JPP140-*rcsA*-F	GATTTGTGCAGTTACAC CCG	
JPP141-*rcsA*-R	GATAGCGAGTTCGTCAA CGG	
JPP138-*yjbG*-F	GTGGTTTACCCTGATGG ACG	
JPP139-*yjbG*-R	AGAATGTCGGCATTTAT CGC	
JPP136-*wcaA*-F	ATCCGAAATCCCCTTATT CG	
JPP137-*wcaA*-R	TGCGCAGAAAAATGTCG TAG	
JPP8-*gmd*-F	CAACCCGAAATTTCATCT GC	

JPP9-*gmd-R*	CGCGTTTTCTTTTCAAGA CC	
JPP12-*wza*-F	ACAGAGCACCCTCAAAA TGG	
JPP13-*wza*-R	TGGCGTCAGACATATCA AGC	
JPP249-*rcsB* for plasmid-R	TAAGCGTAGCGCCATCA GGCTG	
JPP250-*rcsB* for plasmid-F	CGCCTGAAAGGGGTGTT TGCC	
delta*csgD*-F	CAGCTGTCAGATGTGCG ATTAAAAAAAGTGGAGTT TCATCGTGTAGGCTGGA GCTGCTTC	
delta*csgD*-R	CTCTGCTGCTACAATCC AGGTCAGATAGCGTTTC ATGGCCCATATGAATAT CCTCCTTAG	
